# A facile method of transforming FGD gypsum to α-CaSO_4_·0.5H_2_O whiskers with cetyltrimethylammonium bromide (CTAB) and KCl in glycerol-water solution

**DOI:** 10.1038/s41598-017-07548-3

**Published:** 2017-08-01

**Authors:** Qingjun Guan, Wei Sun, Yuehua Hu, Zhigang Yin, Changping Guan

**Affiliations:** School of Mineral Processing and Bioengineering, Central South University, Changsha, 410083 China

## Abstract

A facile method to transform flue gas desulfurization gypsum (FGD gypsum) to α- calcium sulfate hemihydrate (α-HH) whiskers with high aspect ratios mediated by cetyltrimethylammonium bromide (CTAB) and KCl in glycerol-water solutions was studied. Addition of KCl facilitated the dissolution of calcium sulfate dihydrate (DH) and created a much higher supersaturation, which could come into being a larger driving force for the phase transformation from DH to α-HH. CTAB as the crystal modifier can significantly promoted 1-D growth of α-HH whiskers along the c axis and the presence of 0.25% CTAB (by weight of FGD gypsum) resulted in the increase of the average aspect ratio of α-HH whiskers from 28.9 to 188.4, which might be attributed to the preferential adsorption of C_16_H_33_(CH_3_)_3_N^+^ on the negative side facets of α-HH crystal.

## Introduction

Flue gas desulfurization gypsum (FGD gypsum), which is mainly composed of CaSO_4_·2H_2_O (DH), is the industrial waste residue produced by limestone-gypsum wet process of flue gas desulfurization technology. The bulk deposition of FGD gypsum can result in not merely large land occupation, but also land and water pollution. How to rationally utilize FGD gypsum has drawn much attention during recent years. Traditionally, the gypsum can be used as the raw material of a cement retarder^1^, or processed to the building gypsum (β- CaSO_4_·0.5H_2_O)^2^ or high-strength gypsum (α- CaSO_4_·0.5H_2_O)^3, 4^. However, all of these products are low value-added. And the emergence of calcium sulfate whiskers (a new type of α- CaSO_4_·0.5H_2_O) with high added value has attracted tremendous interests of researchers due to the excellent performance in thermal stability, chemical resistance and mechanical strength^5–8^. The inexpensive whiskers, which can obviously enhance the mechanical properties of rubbers, plastics, adhesives and papers^9–14^, have great potential to become ideal reinforcing materials.

Much effort has been made to control the crystallization and morphology of α- CaSO_4_·0.5H_2_O (α-HH) prepared by kinds of raw materials to produce high quality whiskers. In general, α-HH whiskers with high aspect ratios are prepared by analytical reagent CaSO_4_·2H_2_O^15–18^ or calcic minerals such as natural gypsum^19^, phosphogypsum^20^ and FGD gypsum^21–24^ in aqueous solutions with elevated temperature under elevated pressure, or by the chemical reaction between CaCl_2_ (AR) and H_2_SO_4_ (AR) in aqueous solutions^25, 26^ or reverse microemulsions^27^ under facile conditions. Meanwhile, in order to improve the aspect ratios of crystal products, crystal modifiers preferentially adsorbed on the side facets of the crystals, tend to be added into the crystallization reaction system. For example, metal ions such as Mg^2+^ ^16, 19^, Cu^2+^ ^28^, Al^3+^ ^29^, can increase the aspect ratios of the whiskers dramatically from dozens to hundreds, and some organic additives, such as cetyltrimethyl ammonium bromide (CTAB)^25, 27^ and ethylene glycol^18^, can also produce α-HH whiskers with the aspect ratio up to hundreds.

However, aiming at preparation of the whiskers with high aspect ratios from calcic minerals, previous studies mainly proceeded with the autoclave method at high temperature and pressure. Few research was carried out to explore the preparation of α-HH whiskers with high aspect ratios by use of FGD gypsum under facile conditions. Recent scientific researches find that because the alcohol aqueous solution has a low water activity, the transformation from DH to α-HH can be realized in alcohol water solution under mild conditions^30, 31^. Compared with the autoclave method, this process with the advantages of mild reaction conditions and no corrosion to equipment is more favorable to the continuous industrial production. Although the transition from DH to α-HH is kinetically unfavorable in alcohol water solution, addition of small amount of non-lattice cations (such as K^+^, Na^+^, Mg^2+^ and Zn^2+^) can significantly shorten the transition time and improve the transition efficiency^32^.

In this paper, we introduced a facile method to prepare α- CaSO_4_·0.5H_2_O (α-HH) whiskers with small diameters and high aspect ratios by use of FGD gypsum in glycerol-water solutions. In the preparation process, CTAB and KCl were used as the crystal modifier and phase transformation accelerator, respectively, and the corresponding mechanism was studied.

## Experimental

### Materials

The FGD gypsum, of which the chemical compositions were given in Table 1, was received from Panzhihua Iron & Steel Co., Ltd., Sichuan Province, China. Glycerol, KCl and Cetyltrimetyl Ammonium Bromide (CTAB) were purchased from Sinopharm Chemical Reagent Co., Ltd., Shanghai, China.

**Table 1:** The chemical composition of the FGD gypsum (wt%).

CaO	SO_3_	H_2_O	SiO_2_	Fe_2_O_3_	Al_2_O_3_	MgO	PbO	K_2_O	Total
36.87	42.63	14.95	0.73	0.31	0.31	0.11	0.06	0.04	96.01

### Experimental procedure

#### Pretreatment of FGD gypsum

The gypsum was calcinated at 100 °C for 5 h in a drying oven, then mixed with enough deionized water and stirred (100 rpm) at room temperature for half hours. After complete hydration, the gypsum was filtrated and dried at 60 °C.

#### Preparation of α-HH whiskers

Glycerol (65 wt%)-water solutions with a certain amount of KCl and CTAB were firstly added into a three-necked flask equipped with a reflux condenser on top of it, and the solution was stirred with a magnetic stirrer at a constant rate of 150 rpm and preheated to 90 °C in an oil bath. Then 50 g FGD gypsum (10 wt % solid content) after pretreated was added into the reactor. During the reaction, 20 mL hot slurry was withdrawn at certain time intervals. Half of the sample was filtrated immediately, washed three times with boiling water and then rinsed once with ethanol before it was dried at 60 °C for 2 hours in an oven. The other 10 mL slurry was filtered by a syringe filter with a 0.2 μm cellulose membrane for Ca^2+^ concentration determination.

### Characterization

The chemical compositions of FGD gypsum were investigated by using X-ray fluorescence spectroscopy (XRF, Axios mAX, PANalytical B.V., Netherlands). The structures of the samples were determined by X-ray diffraction (XRD D8 Advanced, Bruker, Germany) using Cu Kα radiation (λ = 1.54178 Å), with a scanning rate of 5° min^−1^ and a scanning 2θ range of 5° to 70°. The morphology of the samples were characterized with the field-emission scanning electron microscopy (SEM, JSM-6490LV, JEOL, Japan) and the high resolution transmission electron microscopy (HRTEM, JEM-2100F, JEOL, Japan) equipped with the selected area electron diffraction (SAED). The surfaces of the whiskers were analyzed by X-ray photoelectron spectroscopy (XPS, ESCALAB 250Xi, Thermo Fisher, USA) with a Al Kα photon energy of 1486.6 eV. C 1 s peak at 284.8 eV, which is related to the carbon adsorbed on the surface during the exposure of the samples to the ambient atmosphere, was used as reference for all spectra. The interaction between CTAB and the crystal surfaces was analyzed by Flourier Translation Infrared Spectroscopy (FT-IR, IRAffinity-1, Shimadzu, Japan) with a resolution of 4 cm^−1^ over the frequency range of 400–4000 cm^−1^. For thermogravimetry and differential scanning calorimetry (TG-DSC, STA 8000, PerkinElmer, USA), 10 mg sample after dried was put into an Al_2_O_3_ crucible with a lid under temperature programming from 60 °C to 400 °C at a heating rate of 10 °C/min under nitrogen gas atmosphere. The concentration of Ca^2+^ in the filtrate was determined by the inductively coupled plasma-atomic emission spectrometry (ICP-AES, PS-6, Baird, USA). The average lengths and aspect ratios of the whiskers for each sample were counted up about 150 whiskers by Image-Pro plus 6.0 from SEM images with the magnifications of 500–4000.

## Results and Discussion

### Influence of KCl on the rate of phase transformation from FGD gypsum to α-HH

**Figure 1:**
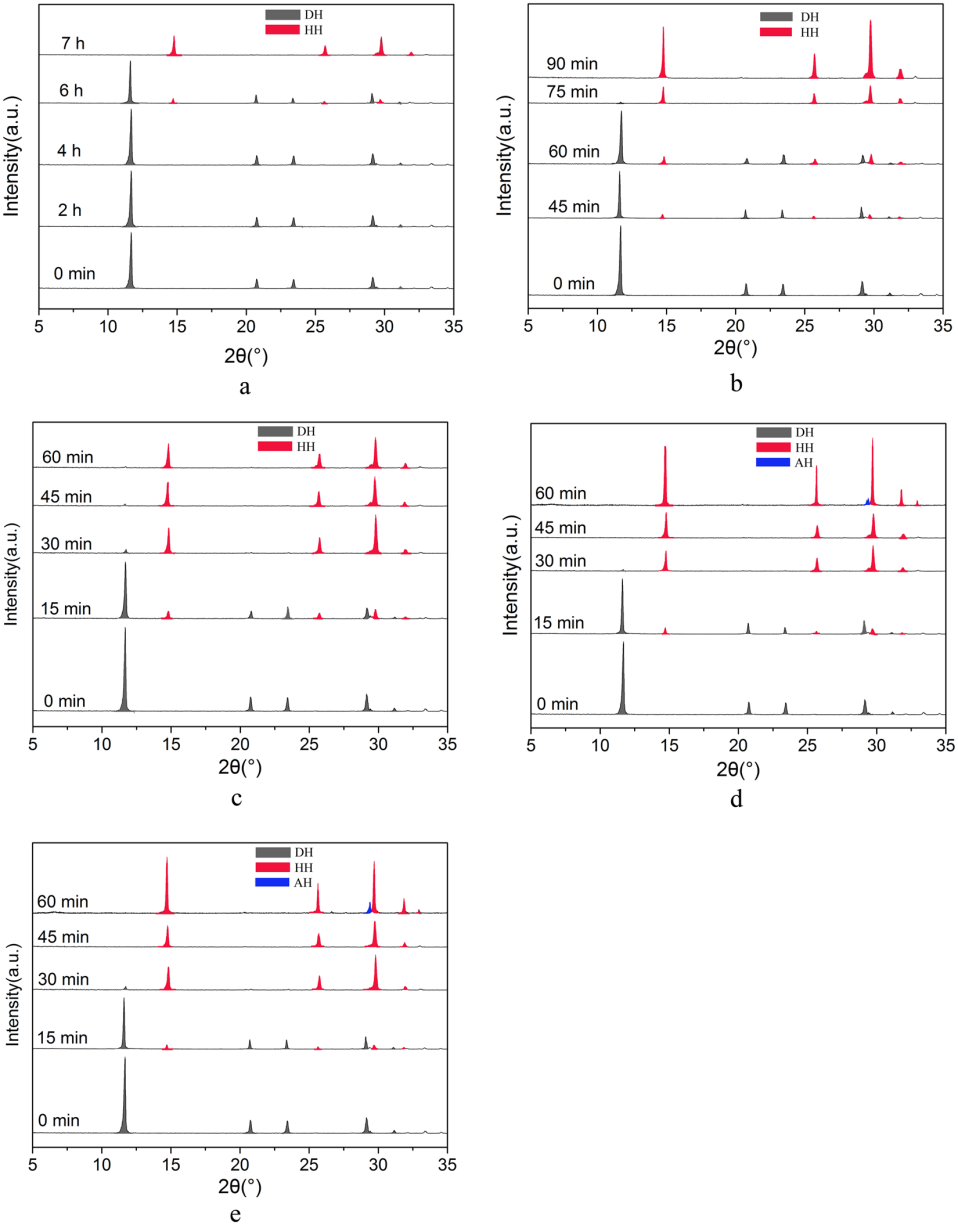
XRD patterns of the crystal products at interval time during the phase transformation in the presence of different KCl concentration in glycerol (65 wt%)-water solutions at 90 °C ((**a**) 0; (**b**) 1%; (**c**) 3%; (**d**) 5%; (**e**) 7%).

In the process of preparation of α-HH whiskers by using FGD gypsum under facile conditions, K^+^ was used to accelerate the phase transformation rate^32^. As shown in Fig. 1, XRD patterns of the crystal products withdrawn at interval time were used to track the phase transformation process. The diffraction peaks of α-HH are at 2θ = 14.72°, 25.64°, 29.70°, 31.86° and FGD gypsum (DH) at 2θ = 11.61°, 20.69°, 23.35° and 29.08°.

Without KCl (Fig. 1a), the transformation from FGD gypsum to α-HH began at 360 min and completed within 420 min. When 1% KCl (by weight of FGD gypsum) (Fig. 1b) was added into the mixed solutions, the transformation started at 45 min and completed at 90 min. When KCl concentration was increased to 3% (Fig. 1c), the gypsum began to dehydrate at 15 min and the phase transformation completed within 1 h. Further increasing KCl concentration to 5% (Fig. 1d) and 7% (Fig. 1e), the time of complete transformation from FGD gypsum to α-HH decreased to 45 min. And as KCl concentration increased, α-HH generated by FGD gypsum would continue to dehydrate and be more easily transformed into anhydrous calcium sulfate (AH).

In order to further illustrate the difference of phase transformation rate with different KCl concentration (1%, 3% and 5% KCl), the crystal water content of samples withdrawn at certain time intervals was measured by TG-DSC technology, and the results are as follows.

**Figure 2:**
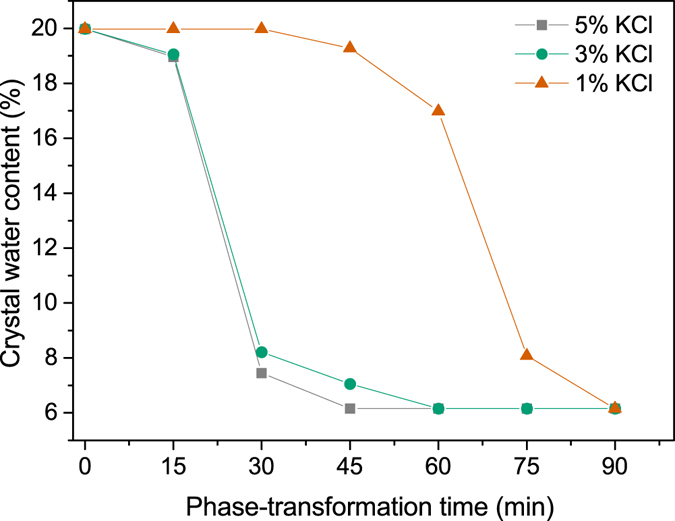
Influence of KCl on the time of phase transformation from FGD gypsum to α-HH in glycerol (65 wt%)-water solutions with different KCl concentration at 90 °C.

The water content of samples withdrawn at certain time intervals was cited to indicate whether FGD gypsum (20.93 wt%) has been completely transformed into α-HH (6.21 wt%). Figure 2 shows the water content change of samples prepared in glycerol (65%)-water mixtures with 1%, 3% and 5% KCl, respectively. As depicted in Fig. 2, in the presence of 1% KCl, the transformation started between 30 min and 45 min, and the crystal water content generally decreased over time and reached 6.21 wt% at 90 min, suggesting a complete transformation. When KCl concentration was increased to 3%, the FGD gypsum began to dehydrate between 0 and 15 min and the complete transformation needed 60 min. Further increasing KCl concentration to 5%, the water content of samples withdrawn at the same time intervals was lower than that of 3%, implying that the phase transformation rate with 5% KCl was faster. And the complete transformation only needed 45 min.

The promoting effect of KCl on the transformation rate should be attributed to the supersaturation (S_HH_) of α-HH in the solution. A higher supersaturaion provided a larger driving force for α-HH nucleus formation, resulting in a shorter time of the phase transformation from FGD gypsum to α-HH. S_HH_ is defined as:

1 $${S}_{HH}=\frac{{a}_{C{a}^{2+}}\cdot {a}_{S{O}_{4}^{2-}}\cdot {a}_{{H}_{2}O}^{0.5}}{{K}_{SP,HH}}=\frac{{c}_{C{a}^{2+}}\cdot {\gamma }_{C{a}^{2+}}\cdot {c}_{S{O}_{4}^{2-}}\cdot {\gamma }_{S{O}_{4}^{2}}\cdot {a}_{{H}_{2}O}^{0.5}}{{K}_{SP,HH}}$$

where a, c, γ and K_sp, HH_ are the activity, ion concentration, activity coefficient and thermodynamic equilibrium constant, respectively. In the research, the ratio of the supersaturations with K^+^ ions to that without K^+^ ions was used to illustrate the influence of K^+^ ions on the supersaturations of α-HH:

2 $$\begin{array}{rcl}\frac{{S}_{HH}}{{S}_{HH}^{0}} & = & \frac{{c}_{C{a}^{2+}}\cdot {\gamma }_{C{a}^{2+}}\cdot {c}_{S{O}_{4}^{2-}}\cdot {\gamma }_{S{O}_{4}^{2-}}\cdot {a}_{{H}_{2}O}^{0.5}}{{K}_{SP,HH}}/\frac{{c}_{C{a}^{2+}}^{0}\cdot {\gamma }_{C{a}^{2+}}^{0}\cdot {c}_{S{O}_{4}^{2-}}^{0}\cdot {\gamma }_{S{O}_{4}^{2-}}^{0}\cdot {a}_{{H}_{2}O}^{{0.5}^{0}}}{{K}_{SP,HH}}\\  & = & \frac{{c}_{C{a}^{2+}}\cdot {\gamma }_{C{a}^{2+}}\cdot {c}_{S{O}_{4}^{2-}}\cdot {\gamma }_{S{O}_{4}^{2-}}}{{c}_{C{a}^{2+}}^{0}\cdot {\gamma }_{C{a}^{2+}}^{0}\cdot {c}_{S{O}_{4}^{2-}}^{0}\cdot {\gamma }_{S{O}_{4}^{2-}}^{0}}\end{array}$$

where 0 represents the experimental data without K^+^ ions. The concentration of Ca^2+^ and SO_4_^2−^ deriving from the dissolution of FGD gypsum are equal. Because the temperature was fixed at 90 °C and the concentration of KCl was much lower than that of glycerol, water activity and K_sp, HH_ could be considered as constant.

**Figure 3:**
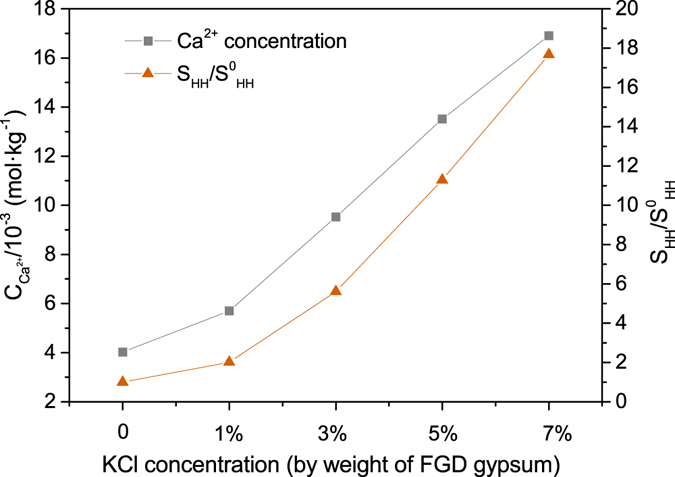
Influence of KCl on Ca^2+^ concentration and S_HH_/S^0^_HH_ in glycerol (65 wt%)-water solutions at 90 °C.

The ionic activity coefficient was calculated by Debye-Hückel law^33^:

3 $$\mathrm{log}\,{\gamma }_{i}=\frac{-A{z}_{i}^{2}{I}^{0.5}}{1+B{a}_{i}{I}^{0.5}}$$

where I, z_i_ and a_i_ are the ionic strength, ionic charge and ion size parameter, respectively. A, B and C are the Debye-Hückel constants. A and B are given by^34^:

4 $$A=\frac{1.8247\times {10}^{6}{\rho }^{0.5}}{{({\varepsilon }_{r}T)}^{1.5}}k{g}^{0.5}\cdot mo{l}^{-0.5}$$

5 $$B=\frac{50.2901\times {10}^{8}{\rho }^{0.5}}{{({\varepsilon }_{{\rm{r}}}T)}^{0.5}}k{g}^{0.5}\cdot mo{l}^{-0.5}\cdot c{m}^{-1}$$

where ρ, ε_r_ and T stand for the density, relative permittivity of the solution, and the thermodynamic temperature, respectively. Since ρ = 1.176 g/cm^3^ and ε_r_ = 58.283 in glycerol (65 wt%)-water solution at 90 °C, A = 0.6427 kg^0.5^·mol^−0.5^ and B = 3.749E + 07 kg^0.5^·mol^−0.5^·cm^−1^ ^32^. And the results are shown in Fig. 3.

As depicted in Fig. 3, as KCl concentration increased, Ca^2+^ concentration and S_HH_/S^0^_HH_ experienced an upward trend. And when KCl concentration increased from 0 to 7%, Ca^2+^ concentration grew from 4.02 × 10^−3^ to 1.69 × 10^−2^ mol·kg^−1^ and S_HH_/S^0^_HH_ from 1.00 to 17.67. It’s clear that addition of KCl facilitated the dissolution of FGD gypsum and provided more available lattice ions, which created a much higher supersaturation and thus came into being a larger driving force for the phase transformation.

### Influence of CTAB on the morphology of α-HH whiskers

**Figure 4:**
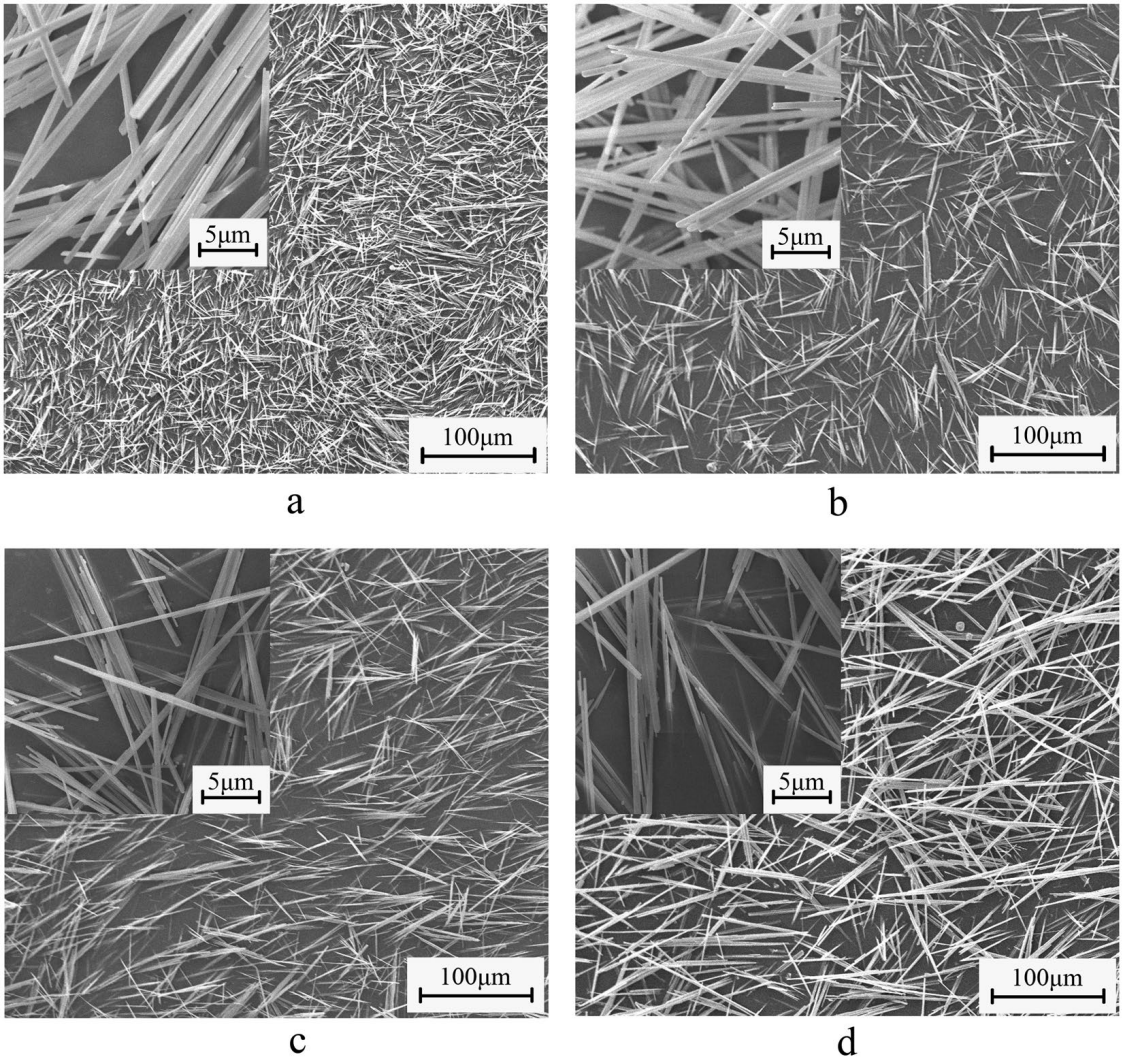
Influence of CTAB on the morphology of α-HH whiskers prepared in glycerol (65 wt%)-water solutions with 5% KCl (by weight of FGD gypsum) at 90 °C for 45 min ((**a**) 0; (**b**) 0.05%; (**c**) 0.15%; (**d**) 0.25%).

**Figure 5:**
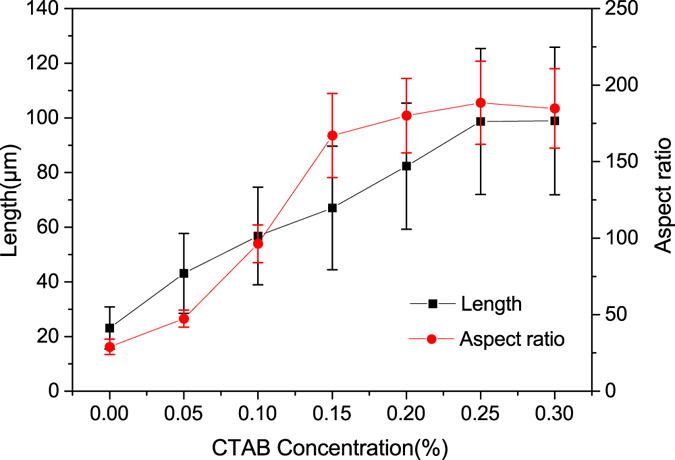
Average length and aspect ratio of α-HH whiskers prepared with different CTAB concentrations in glycerol (65 wt%)-water solutions with 5% KCl (by weight of FGD gypsum) at 90 °C for 45 min.

The morphology, average lengths and aspect ratios of α-HH whiskers formed with different CTAB concentration (by weight of FGD gypsum) in glycerol-H_2_O solution with 5% KCl at 90 °C for 45 min are shown in Figs 4 and 5. In the absence of CTAB (Fig. 4a), the crystal products with a diameter of 0.4–1.6 μm, an average length of 23.1 μm and aspect ratio of 28.9 were formed. When CTAB concentration was lower than 0.25%, the average lengths of the crystals increased and the average diameters decreased gradually with CTAB concentration increasing. Hence the aspect ratios underwent a significant increase. With an increase in CTAB concentration from 0.05% to 0.15%, the average aspect ratios enhanced from 47.5 to 167.1. And when the CTAB concentration reached to 0.25%, the average aspect ratio of α-HH whiskers with a diameter of 0.2–0.8 μm and an average length of 98.7 μm was up to 188.4. Continuing increasing the concentration of CTAB, the length of the crystal products rose unobviously and the diameter underwent a slight increase, which led to a slight decrease in the aspect ratio. The cause of this phenomenon might probably be the influence of high CTAB concentration on the solute diffusion. According to Song’s research^25^, the diffusion coefficient of the solute decreases with the increase of CTAB concentration, indicating that the solute diffusion is prohibited by CTAB. Thus the mass of solute delivered from the solution to crystal surface decreases simultaneously, inhibiting the growth rate of calcium sulfate whisker and resulting in the decrease in the aspect ratio.

**Figure 6:**
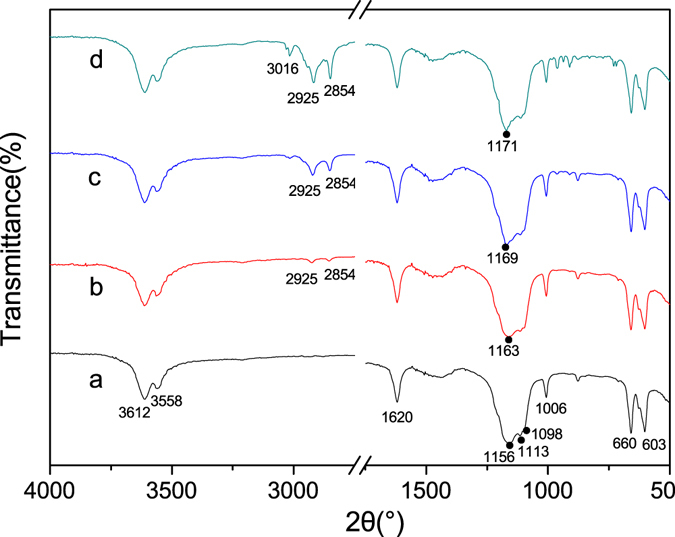
FT-IR spectra of α-HH whiskers prepared with different CTAB concentrations in glycerol (65 wt%)-water solutions with 5% KCl (by weight of FGD gypsum) at 90 °C for 45 min ((**a**) 0; (**b**) 0.05%; (**c**) 0.15%; (**d**) 0.25%).

To confirm the interactions between CTAB and the crystal surfaces of the α-HH crystals, the FT-IR spectra were explored as shown in Fig. 6. The peaks at 3612, 3558, and 1620 cm^−1^ should be assigned to the vibration of O-H. The triple peaks at 1156, 1113, and 1098 cm^−1^ should be assigned to the asymmetric stretching vibration of υ_3_ SO_4_^2−^. And as CTAB concentration rose, the adsorption peak at 1156 cm^−1^ underwent gradually red shifts (1163 cm^−1^ in line b, 1169 cm^−1^ in line c, and 1171 cm^−1^ in line d), implying the strong interaction between CTAB and SO_4_^2−^ group of α-HH^25, 27^. The peak at 1006 cm^−1^ should be assigned to the distorted symmetric stretching vibration of υ_1_ SO_4_^2−^, and the peaks at 660 and 603 cm^−1^ should be indexed to the υ_4_ SO_4_^2−^ stretching. The two adsorption peaks at 2925 and 2854 cm^−1^ in line b, c and d should be attributed to the asymmetric and symmetric stretching vibrations of CH_2_. And the stretching vibrations were gradually enhanced in intensity with an increase of CTAB content, which all indicated the adsorption of CTAB on the surfaces of α-HH crystals.

**Figure 7:**
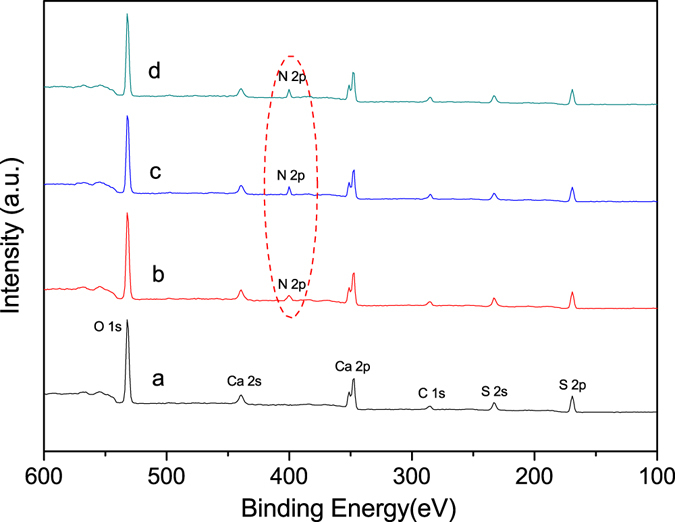
XPS wide scan of α-HH whiskers prepared with different concentration of CTAB in glycerol (65 wt%)-water solutions with 5% KCl (by weight of FGD gypsum) at 90 °C for 45 min ((**a**) 0; (**b**) 0.05%; (**c**) 0.15%; (**d**) 0.25%).

XPS was used to characterize the surface adsorption of CTAB on the crystal products. Figure 7 shows the typical XPS wide-scan spectra of α-HH whiskers formed with different concentration of CTAB. The existence of N 2p spectra at 400 eV in line b, c and d indicated the presence of CTAB in the surface of the hydrothermal products.

**Figure 8:**
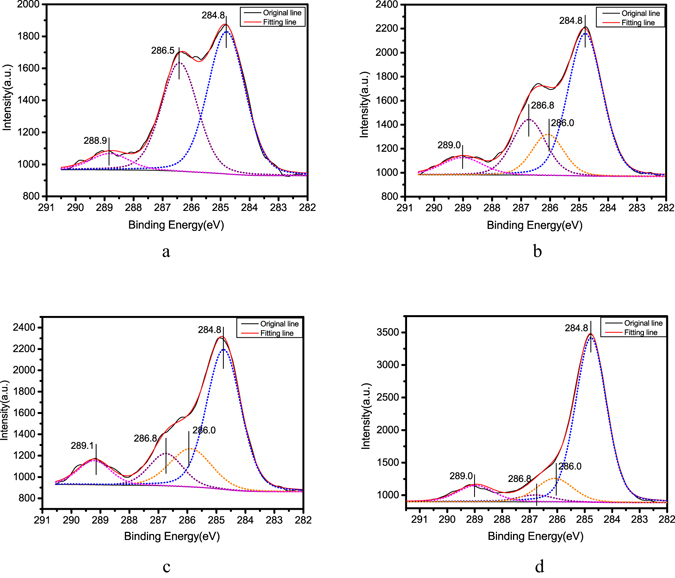
C1s XPS core-level spectra of α-HH whiskers prepared with different concentration of CTAB in glycerol (65 wt%)-water solutions with 5% KCl (by weight of FGD gypsum) at 90 °C for 45 min ((**a**) 0; (**b**) 0.05%; (**c**) 0.15%; (**d**) 0.25%).

The XPS spectra of C1s core level from all four α-HH whisker samples are shown in Fig. 8. The C 1 s spectra of α-HH crystals prepared without CTAB as shown in Fig. 8a could be fitted with three components: The major component at 284.8 eV should be attributed to the majority of carbon contamination on the crystal surface^35^. The component located at 286.5 eV should be associated with C-O in the glycerol^35–38^, implying the adsorption of trace amount of glycerol on the crystal surfaces^18, 39, 40^. And the high binding energy component of C1s at 288.9 should be derived from the carbonate (CO_3_^2−^) of the limestone existed in FGD gypsum^41, 42^. The C 1 s spectra of α-HH whiskers formed with different CTAB concentration (Fig. 8b–d) could be fitted with four components. The C1s peaks at 284.8 eV should be attributed to the carbon atoms in the hydrocarbon chain of CTAB^36, 43^, as well as the majority of carbon contamination on the crystal surface. And the peaks at 286.0 eV should have arisen from the carbon atoms bonded to nitrogen in CTAB^44–46^, which demonstrated the adsorption of the modifier on the crystal surface. Compared to the C-O peak of C1s XPS spectra from α-HH crystals prepared without CTAB, the C-O peaks of C1s XPS spectra from α-HH whiskers formed in the presence of CTAB shifted to the higher binding energy, up to 286.8 eV, which might result from the interaction between glycerol and CTAB adsorbed on the crystal surfaces.

As shown in Fig. 8, the intensity of the major component (284.8 eV) of C1s XPS spectra enhanced gradually with the increase of CTAB concentration, which also proved the adsorption of CTAB on the whisker surface.

Table 2 shows the surface atomic distribution of carbon and nitrogen of α-HH whiskers formed in the presence of different CTAB concentration. From Table 2, we can see that as the increase of CTAB concentration, the surface atomic concentration of N and C generally enhanced, demonstrating adsorption of CTAB on the crystal surfaces.

**Table 2:** C and N surface atomic concentration of α-HH whiskers prepared with different CTAB concentration in glycerol (65 wt%)-water solutions with 5% KCl (by weight of FGD gypsum) at 90 °C for 45 min.

Sample	a	b	c	d
AtomicConcentration (%)				
C	8.98	9.23	11.74	12.06
N	0	0.26	0.40	0.59

**Figure 9:**
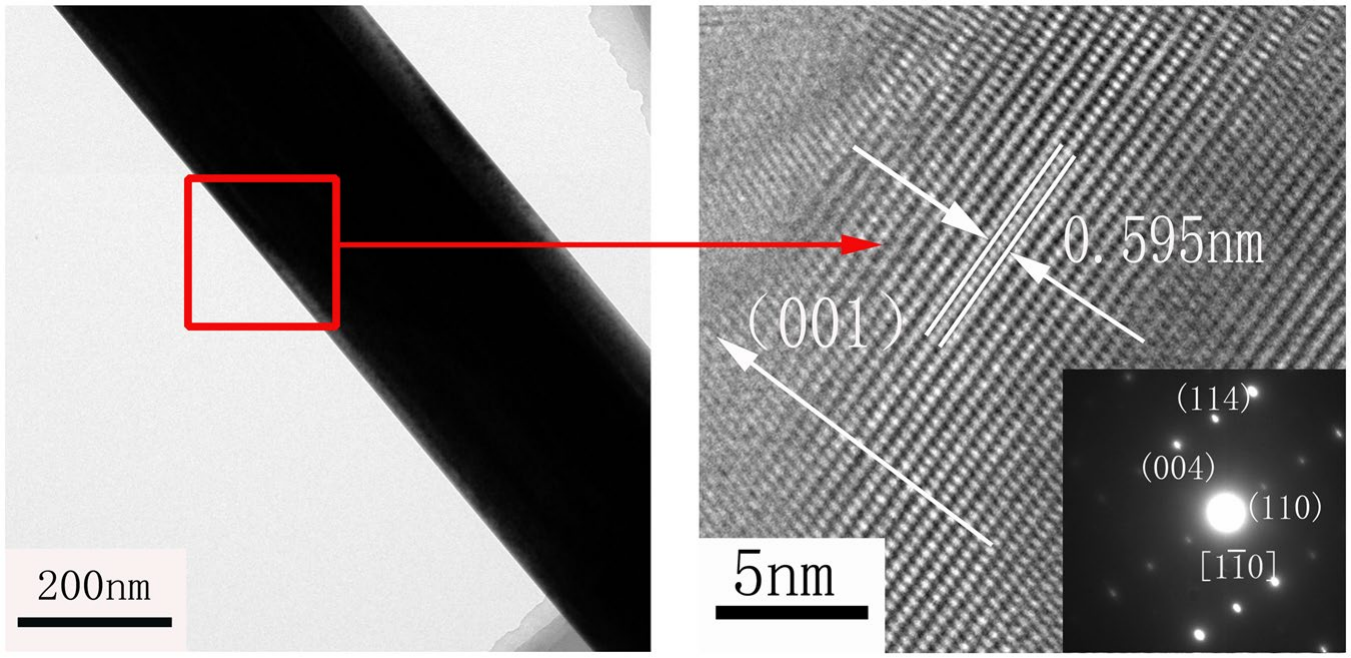
TEM and HRTEM images and SAED pattern of α-HH whiskers prepared in the presence of 0.25% CTAB in glycerol (65 wt%)-water solutions with 5% KCl (by weight of FGD gypsum) at 90 °C for 45 min.

TEM, high-resolution TEM (HRTEM) and SAED were performed to explore the morphology and structure of the crystal products further. As shown in Fig. 9, the interplanar spacing of the lattice fringes along the whiskers‘ growth direction was 0.595 nm, corresponding to the (002) plane (d(002) = 0.599 nm) of α-HH, which indicated that the crystals grow along the (001) direction. The electronic diffraction spots in the SAED pattern could be indexed to the [1$\bar{1}$0] zone axis of α-HH, further confirming the preferential growth of the crystals along the (001) direction.

**Figure 10:**
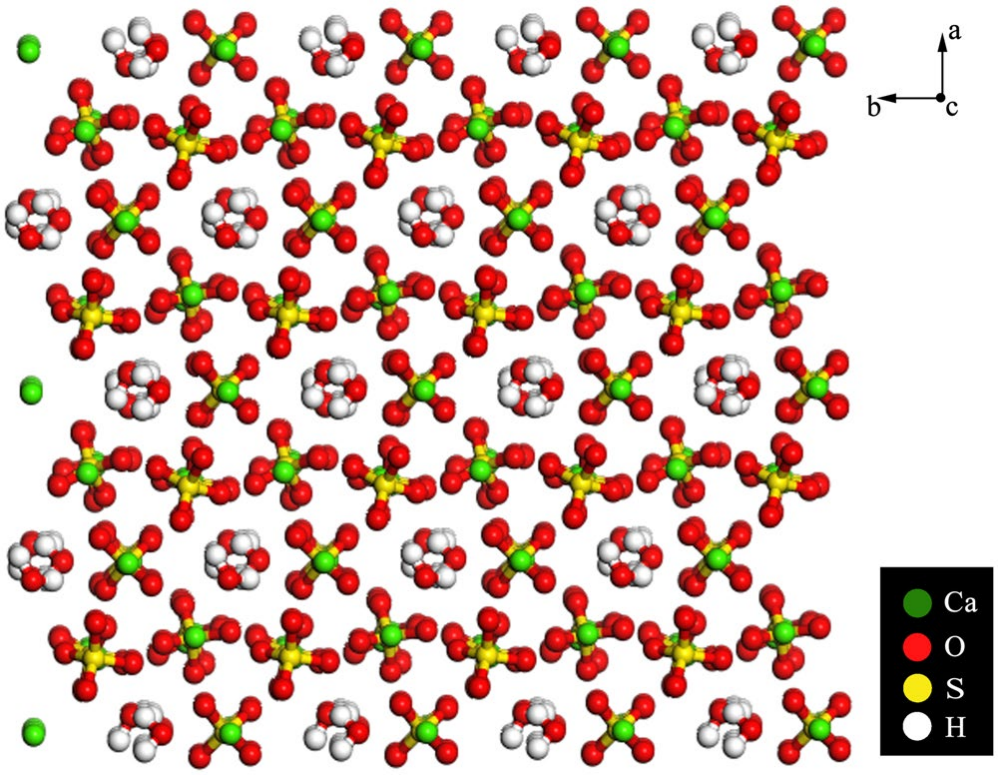
The crystal structure of α-HH lattice along c axis.

**Figure 11:**
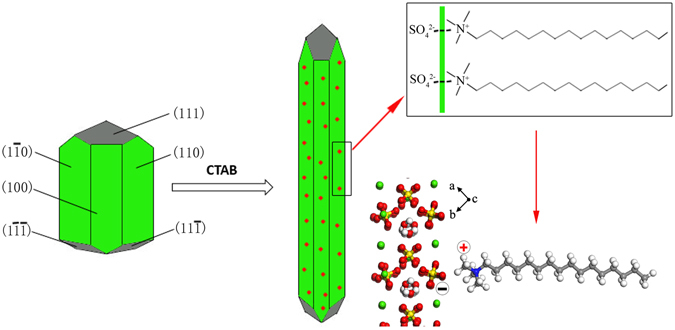
Schematic diagram of adsorption of CTAB on the surfaces of α-HH crystal.

As shown in Fig. 10, α-HH lattices are composed of repeating, ionically bonded Ca and SO_4_ atoms in chains of -Ca -SO_4_-Ca -SO_4_-. And SO_4_ is a tetrahedral structure in which each S atom is covalently bonded to four O atoms^47, 48^. The chains’ structure may account for the fact that α-HH normally grows in the 1D shape. These chains are hexagonally and symmetrically arranged and form a framework parallel to the c axis. And along the c axis there exist continuous channels with a diameter of about 4.5 Å, where one water molecule is attached to every two calcium sulfate molecules^49^. The crystal structure makes the distribution of Ca^2+^ denser on the top facets of {111} and the distribution of SO_4_^2−^ denser on the side facets of {110} and {100}, which makes {111} facets positively charged and {110} and {100} facets negatively charged^47, 49^. Therefore, the positive C_16_H_33_(CH_3_)_3_N^+^ is adsorbed more easily on the side facets of α-HH crystal, which promotes the 1-D growth of α-HH whiskers along the c axis, as shown in Fig. 11.

## Conclusion

α-HH whiskers with high aspect ratios could be prepared by using FGD gypsum in glycerol-water solutions with CTAB and KCl under facile conditions. KCl played a key role in speeding up the transformation rate while CTAB acted as a crystal modifier to promote 1-D growth of α-HH along the c axis. The presence of 5% KCl could result in a significant increase in the supersaturation of α-HH, which dramatically reduced the time of complete phase transformation from 7 h to 45 min. And as KCl concentration increased, α-HH generated by FGD gypsum would continue to dehydrate and be more easily transformed into anhydrous calcium sulfate (AH). The existence of 0.25% CTAB in the crystallization reaction system could lead to a significant increase of the average aspect ratio from 28.9 to 188.4, which might probably be attributed to preferential adsorptioin of positive C_16_H_33_(CH_3_)_3_N^+^ on the side facets of α-HH crystal and promote the 1-D growth of α-HH whiskers along the c axis. And continuing increasing CTAB concentration, the aspect ratios suffered a slight decrease.
